# Supplementary material for: Passive superresolution imaging of incoherent objects
Source: Optica. 2023 Aug 22;10(9):1147–52. doi: 10.1364/OPTICA.493718 (PMC13541222; doi:10.1364/OPTICA.493718)
Supplement: Supplementary file 1 [file optica-10-9-1147-s001.pdf]

Supplemental Document

OPTICA

Passive superresolution imaging of incoherent objects: supplement

Jernej Frank, Alexander Duplinskiy, Kaden Bearne, and A. I. Lvovsky*

Department of Physics, University of Oxford, Oxford OX1 3PU, UK
*alex.lvovsky@physics.ox.ac.uk

This supplement published with Optica Publishing Group on 22 August 2023 by The Authors under the terms of the Creative Commons Attribution 4.0 License in the format provided by the authors and unedited. Further distribution of this work must maintain attribution to the author(s) and the published article's title, journal citation, and DOI.

Supplement DOI: https://doi.org/10.6084/m9.figshare.23808471

Parent Article DOI: https://doi.org/10.1364/OPTICA.493718

Passive superresolution imaging of incoherent objects: supplemental document

1. DERIVATION OF EQS. (1) AND (2)

Consider an object with the electric field distribution in the object plane given by $E(\vec{\rho})$. Imaging the object with the objective lens will result in the electric field distribution in the image plane that is the convolution of E and the point spread function ψ:

$$\tilde{E}(\vec{r}) = \int E(\vec{\rho})\psi(\vec{r}-\vec{\rho})\vec{\rho}. \tag{S1}$$

Heterodyne detection of the image field with the local oscillator prepared in a Hermite-Gaussian mode $\phi_{mn}(\vec{r})$ will produce the photocurrent

$$J_{mn} = \int \phi_{mn}(\vec{r})\tilde{E}(\vec{r}) = \iint E(\vec{\rho})\psi(\vec{r}-\vec{\rho})\phi_{mn}(\vec{r})\vec{\rho}. \tag{S2}$$

For incoherent objects, we measure the time-averaged power (absolute square) of the heterodyne photocurrent, which is given by

$$P_{mn} = \langle |J_{mn}|^2 \rangle = \iiiint \langle E(\vec{\rho})E^*(\vec{\rho}')\rangle \psi(\vec{\rho}-\vec{r})\psi^*(\vec{\rho}'-\vec{r}')\phi^*_{mn}(\vec{r})\phi_{mn}(\vec{r}')\,\vec{\rho}\vec{\rho}'\vec{r}\vec{r}'. \tag{S3}$$

In the above result, we can recognise $\langle E(\vec{\rho})E^*(\vec{\rho}')\rangle$ as the mutual intensity function. For a fully incoherent object with the intensity distribution $I(\vec{\rho})$, the mutual intensity is

$$\langle E(\vec{\rho})E^*(\vec{\rho}')\rangle = I(\vec{\rho})\delta(\vec{\rho}-\vec{\rho}').$$

Hence

$$P_{mn} = \iiint I(\vec{\rho})\psi(\vec{\rho}-\vec{r})\psi^*(\vec{\rho}-\vec{r}')\phi^*_{mn}(\vec{r})\phi_{mn}(\vec{r}')\,\vec{\rho}\vec{r}\vec{r}'. \tag{S4}$$

The above integral can be written as

$$P_{mn} = \int I(\vec{\rho})\,|[\phi_{mn} * \psi](\vec{\rho})|^2\,, \tag{S5}$$

where $\phi_{mn} * \psi$ is the convolution of the Hermite-Gaussian mode

$$\phi_{mn}(\vec{r}) = \frac{H_k(\frac{x}{\sqrt{2}\sigma})H_k(\frac{y}{\sqrt{2}\sigma})}{\sqrt{2^{m+n}m!n!}}\psi(\vec{r})$$

and the Gaussian PSF

$$\psi(\vec{\rho}) = \frac{1}{\sqrt{2\pi\sigma^2}}e^{-\frac{x^2+y^2}{4\sigma^2}}, \tag{S6}$$

which is assumed to match ϕ_{00}. This convolution is known to equal [1]

$$[\phi_{mn} * \psi]\,(x,y) = \frac{1}{\sqrt{m!n!}}\left(\frac{x}{2\sigma}\right)^m\left(\frac{y}{2\sigma}\right)^n e^{-\frac{x^2+y^2}{8\sigma^2}}, \tag{S7}$$

so we finally get

$$P_{mn} = \frac{1}{m!n!}\int I(x,y)\left(\frac{x}{2\sigma}\right)^{2m}\left(\frac{y}{2\sigma}\right)^{2n} e^{-\frac{x^2+y^2}{4\sigma^2}}\,dxdy. \tag{S8}$$

From these measurements, we can calculate the following sums with even indices p and q,

$$\beta_{pq} = \sum_{\text{even } k,l=0}^{p,q} \left(\frac{k}{2}\right)! \left(\frac{l}{2}\right)! \alpha_{pk}\alpha_{ql} P_{\frac{k}{2}\frac{l}{2}}, \tag{S9}$$

with the α's being the coefficients of Hermite polynomials $H_i(x) = \sum_j^i \alpha_{ij}x^j$. These sums are equal to scalar products between the object intensity distribution and the corresponding Hermite-Gaussian polynomials:

$$\beta_{pq} = \sum_{\text{even } k,l=0}^{p,q} \alpha_{pk}\alpha_{ql} \int I(x,y) \left(\frac{x}{2\sigma}\right)^k \left(\frac{y}{2\sigma}\right)^l e^{-\frac{x^2+y^2}{4\sigma^2}}\, dxdy \tag{S10}$$

$$= \sum_{\text{even } k,l=0}^{p,q} \int I(x,y)\phi_{kl}(x,y)\, dxdy. \tag{S11}$$

Because 2-dimensional Hermite-Gaussian polynomials ϕ_{pq} are orthonormal with weight $2^{p+q+2}p!q!\pi\sigma^2$, we have that β's are the expansion coefficients of $I(x,y)$ into the Hermite-Gaussian basis:

$$I_{\text{even}}(x,y) = \sum_{p,q=0}^{N} \frac{\beta_{pq}\phi_{pq}(x,y)}{2^{p+q+2}p!q!\pi\sigma^2}, \tag{S12}$$

Because the measurements only recover the β's with even indices, we can reconstruct the even component of the object intensity distribution $I(x,y)$. In other words, if we calculate Eq. (1) from the main text including only even m and n, we will reconstruct the distribution $[I(x,y) + I(-x,y) + I(x,-y) + I(-x,-y)]/4$.

Alternatively, we can recover the odd components by measuring in the overcomplete measurement basis, which includes HG mode superpositions

$$\phi_{mn}^{0-6} := \{\phi_{m,n}, \phi^{\pm}_{m,m+1,n,n}, \phi^{\pm}_{m,m,n,n+1}, \phi^{\pm}_{m,m+1,n,n+1}\},$$

where

$$\phi^{\pm}_{m,m',n,n'} := \frac{1}{\sqrt{2}}[\phi_{m',n'} \pm \phi_{mn}]. \tag{S13}$$

By measuring the heterodyne photocurrent powers $P_{mn}^{(0)-(6)}$ corresponding to these mode superpositions, we can compute the integrals

$$Q_{mn} = \int I(x,y) \left(\frac{x}{2\sigma}\right)^m \left(\frac{y}{2\sigma}\right)^n e^{-\frac{x^2+y^2}{4\sigma^2}}\, dxdy. \tag{S14}$$

according to

$$Q_{mn} = \begin{cases} \left(\frac{m}{2}\right)!\left(\frac{n}{2}\right)! P^{(0)}_{\frac{m}{2}\frac{n}{2}} & m,n \text{ even} \\ \left(\frac{m-1}{2}\right)!\left(\frac{n}{2}\right)!\sqrt{\frac{m+1}{8}}\left(P^{(1)}_{\frac{m-1}{2}\frac{n}{2}} - P^{(2)}_{\frac{m-1}{2}\frac{n}{2}}\right) & m \text{ odd}, n \text{ even} \\ \left(\frac{m}{2}\right)!\left(\frac{n-1}{2}\right)!\sqrt{\frac{n+1}{8}}\left(P^{(3)}_{\frac{m}{2}\frac{n-1}{2}} - P^{(4)}_{\frac{m}{2}\frac{n-1}{2}}\right) & m \text{ even}, n \text{ odd} \\ \left(\frac{m-1}{2}\right)!\left(\frac{n-1}{2}\right)!\sqrt{\frac{m+1}{8}}\sqrt{\frac{n+1}{8}}\left(P^{(5)}_{\frac{m-1}{2}\frac{n-1}{2}} - P^{(6)}_{\frac{m-1}{2}\frac{n-1}{2}}\right) & m,n \text{ odd}. \end{cases} \tag{S15}$$

Knowing Q_{mn}, we can evaluate

$$\beta_{pq} = \sum_{m,n=0}^{pq} \alpha_{pm}\alpha_{qn}Q_{mn} \tag{S16}$$

for all p and q, and hence reconstruct $I(x,y)$.

REFERENCES

1. https://en.wikipedia.org/wiki/Weierstrass_transform#Transforms_of_some_important_functions.
